# Supplementary material for: Pitavastatin activates mitophagy to protect EPC proliferation through a calcium-dependent CAMK1-PINK1 pathway in atherosclerotic mice
Source: Commun Biol. 2022 Feb 10;5:124. doi: 10.1038/s42003-022-03081-w (PMC8831604; doi:10.1038/s42003-022-03081-w)

Supplementary Figures and Legends

Supplementary Figure 1

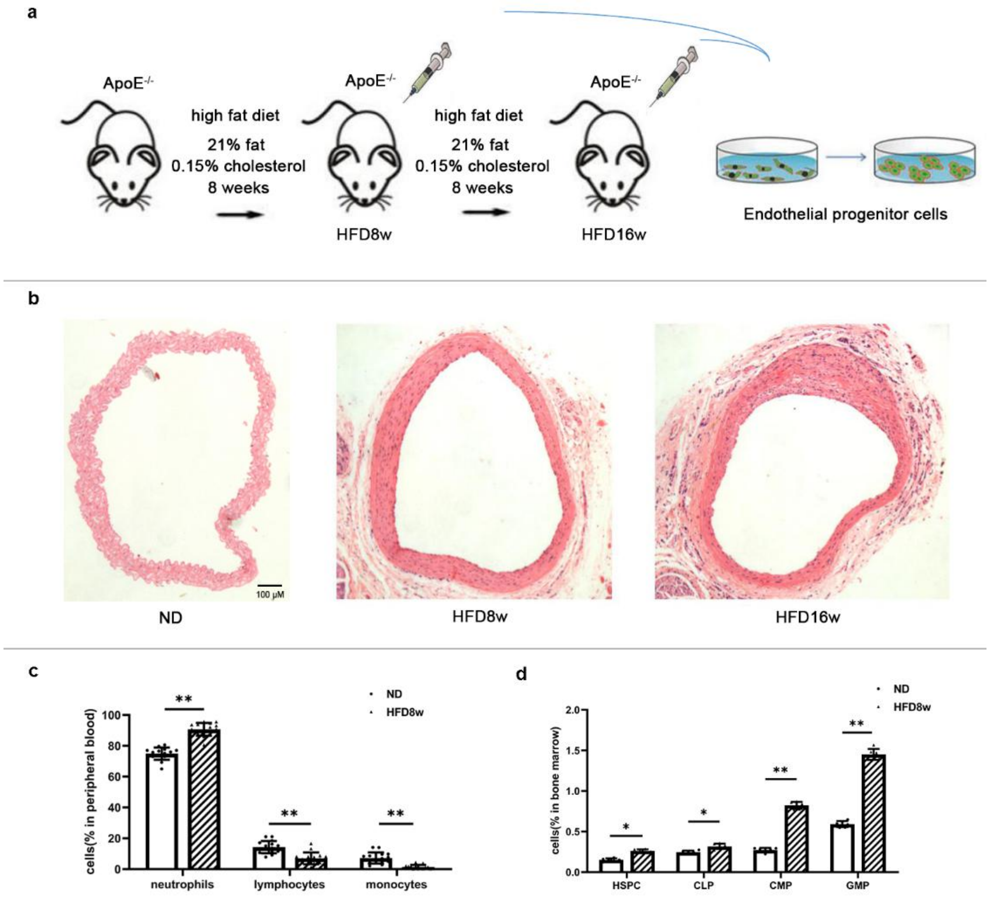

Supplementary Figure 1. Atherosclerotic mice establishment. (a) *ApoE*$^{-/-}$ mice were fed with 21% fat and 0.15% cholesterol for 8 weeks and 16 weeks to establish atherosclerotic mice. We isolated obtained bone marrow-derived mononuclear cells to obtain endothelial progenitor cells. (b) Atherosclerotic lesions from mice aortas were observed to confirm that atherosclerotic mice were established. (c) Quantification of blood neutrophils, lymphocytes and monocytes in control and HFD8w mice. (d) Quantification of HSPC, CLP, CMP, and GMP in control and HFD8w mice.

Supplementary Figure 2

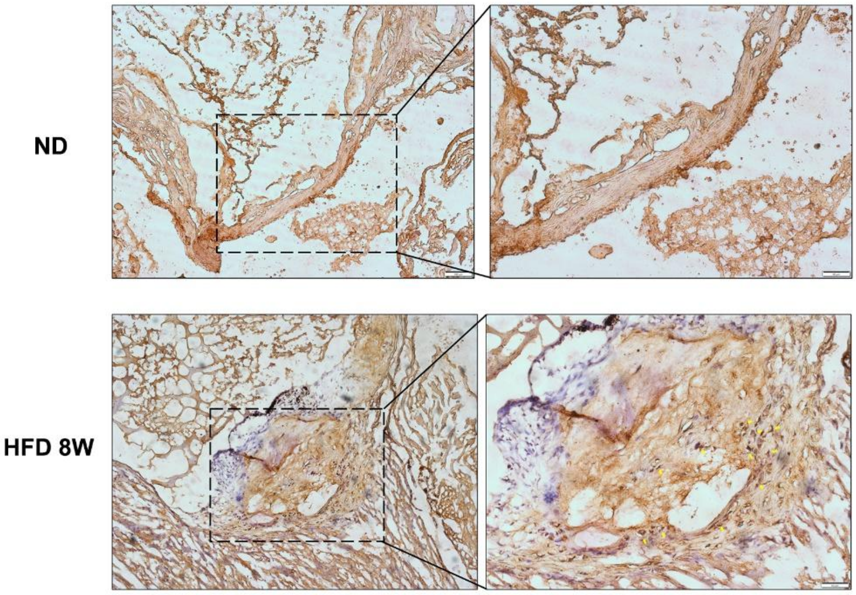

Supplementary Figure 2. CD68-positive macrophages in mice atherosclerotic lesions. Aortas were analyzed by immunohistochemistry. Lesion areas rich in macrophages (yellow arrows) were identified by CD68 (brown) and then analyzed at higher magnification. Bar=50 um. (Aortas were dissected from 3 normal mice and *ApoE*$^{-/-}$ mice fed with high fat diet for 8 weeks).

Supplementary Figure 3

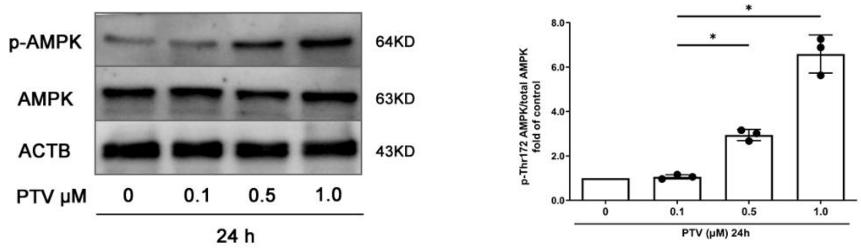

Supplementary Figure 3. PTV increased AMPK phosphorylation. Representative wester blots and quantitative analysis indicated that PTV increased AMPK phosphorylation at Thr^{172} in time-dependent manner.

Supplementary Figure 4

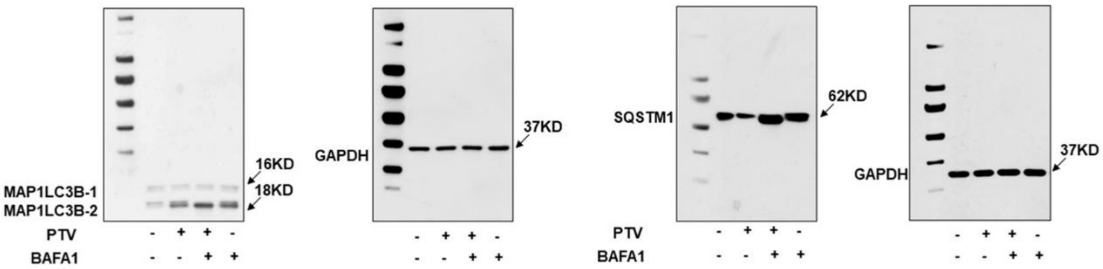

Supplementary Figure 4. EPCs were exposed to BAPTA (10 mM) in either the presence or absence of PTV (0.5 μM). The MAP1LC3B-II turnover and SQSTM1 were analyzed by immunoblot. BAPTA: bis-(aminophenolxy)ethane-N,N,N',N'-tetra-acetic acid. (EPCs were isolated from 3 mice. The figures represented 3 independent experiments).

Supplementary Figure 5

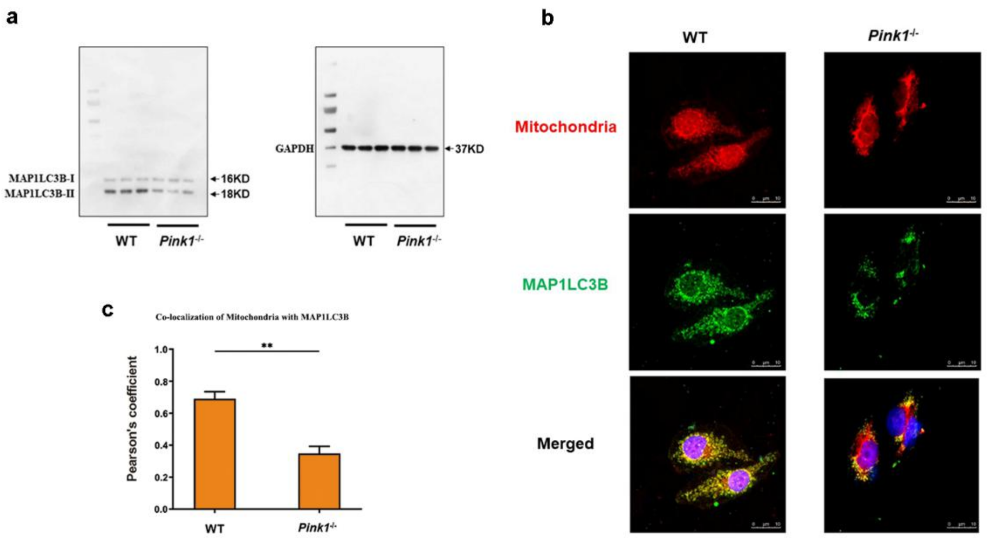

Supplementary Figure 5. *Pink1* deficiency aggravates EPCs mitophagy. EPCs were isolated from WT and *Pink1*$^{-/-}$ mice. (a) Western blots revealed that MAP1LC3B-II turnover decreased in *Pink1* KO mice. (b) Representative LCSM merged images of EPCs and Pearson's overlap coefficient analysis (c) showed that the yellow area decreased in *Pink1*$^{-/-}$ mice group compared with WT group, scale bar: 10 μm. (n=10 cells per group, EPCs were isolated from *ApoE*$^{-/-}$ mice fed with high fat diet for 8 weeks, cells were isolated from 3 mice for 1 experiment and 3 independent experiments were performed, mean ± SD, ***P* < 0.01).

Supplementary Figure 6

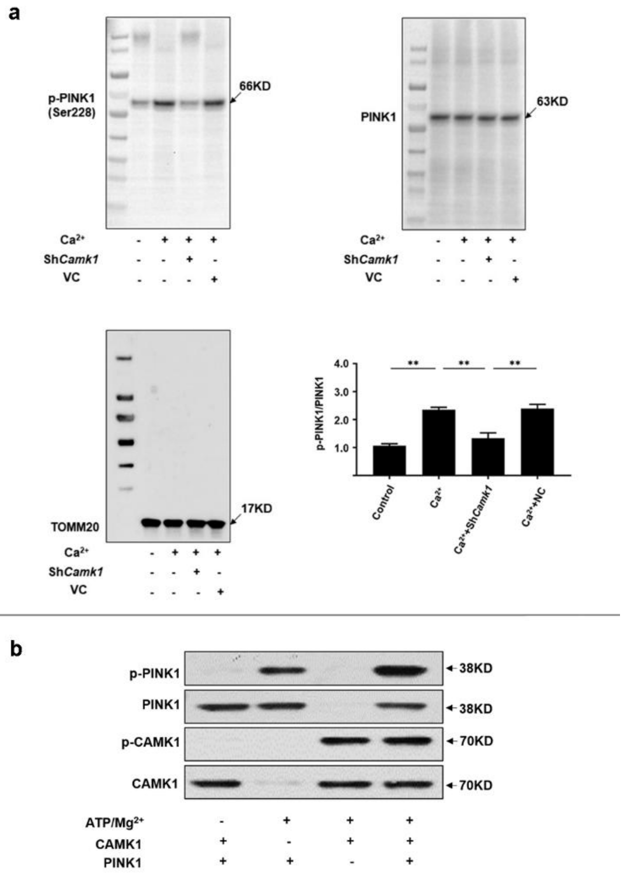

Supplementary Figure 6. CAMK1 contributes to phosphorylation of PINK1 to induce mitophagy. (a) The proportion of p-PINK1 is upregulated in EPCs with high calcium treatment. Representative western blots and quantitative analysis indicated that this effect was significantly reversed when knockdown CAMK1. (b) PINK1 (1μg, MW of recombinant protein, 38 kDa) was incubated in the presence or absence of CAMK1 (25 ng, MW of recombinant protein,70 kDa) for 10 min at 30°C with the following additions: 10 mM $MgCl_2$, 0.2 mM ATP, 1 mM $CaCl_2$, and 1 μM CaM. Reactions were terminated by boiling in SDS–2-ME dissociation solution and analyzed by immunoblot with PINK1 or anti-p-Ser^{228} PINK1 antibody.

Original Western-Blot Scans (Main Article)

Figure. 2a

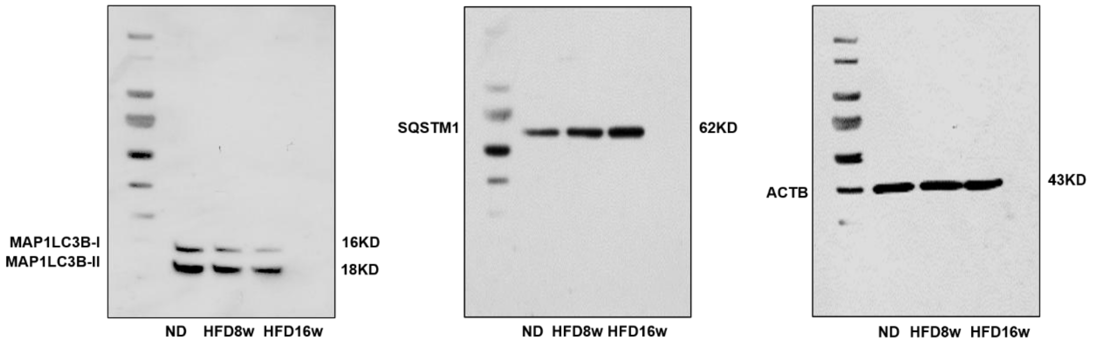

Figure. 2e

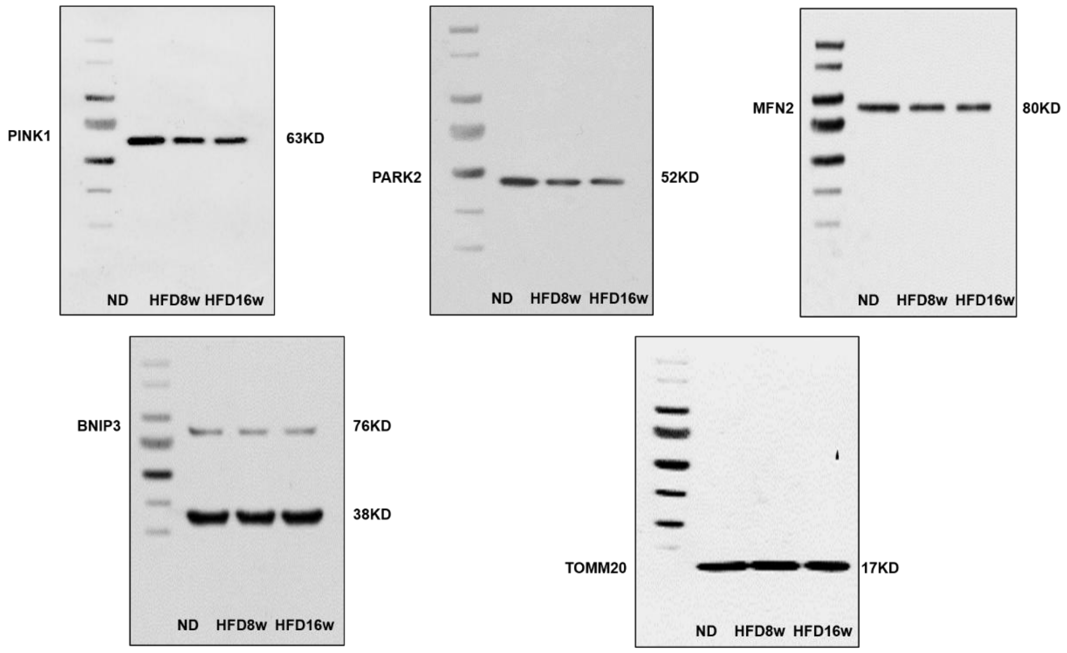

Figure. 3c

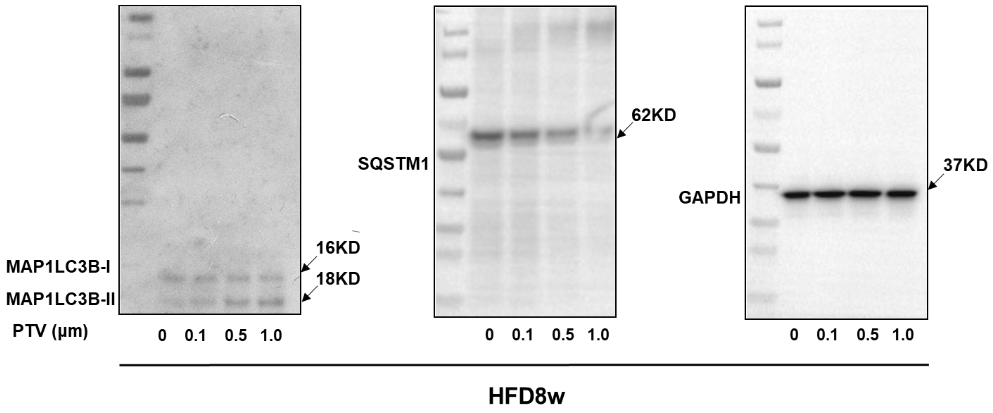

Figure. 3d

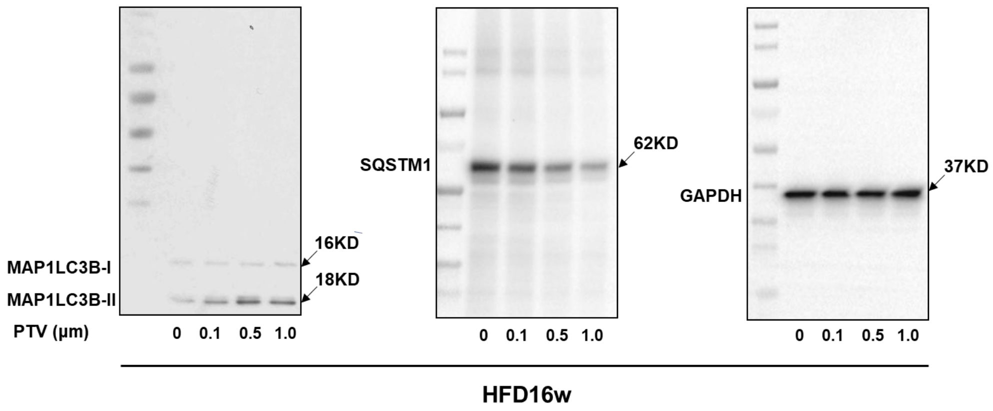

Figure. 4a

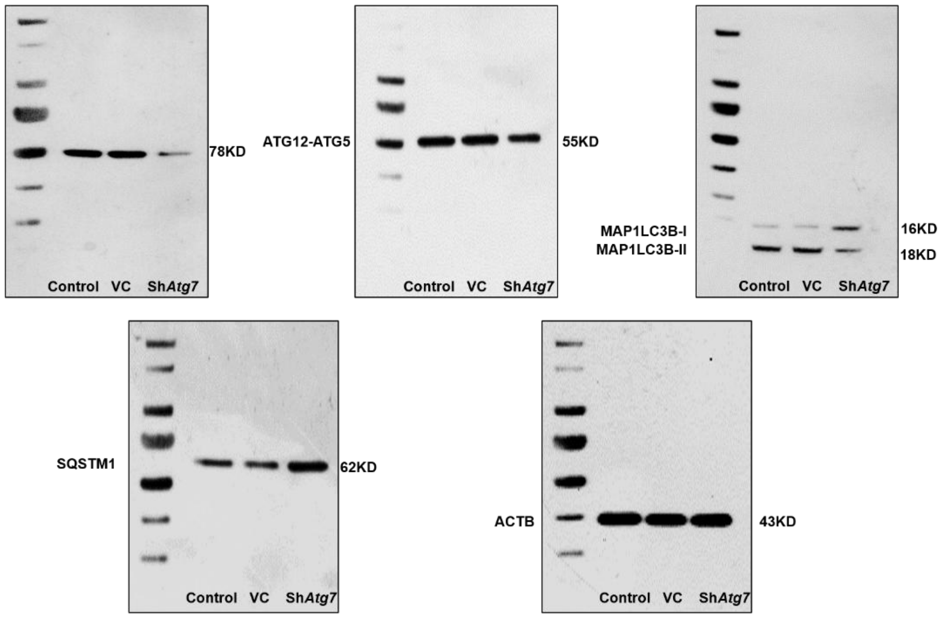

Figure. 4d

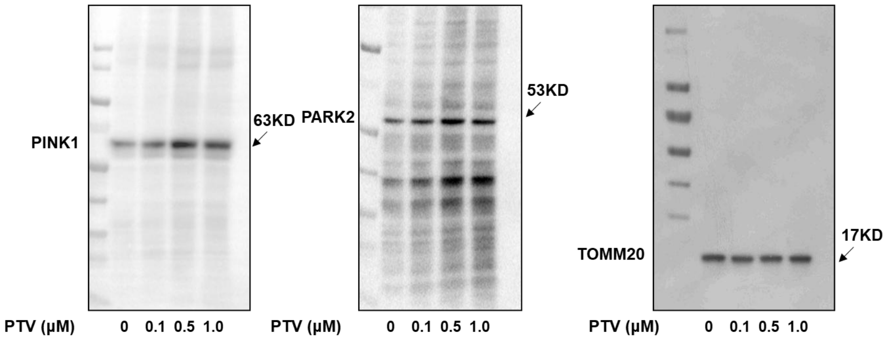

Figure. 5a

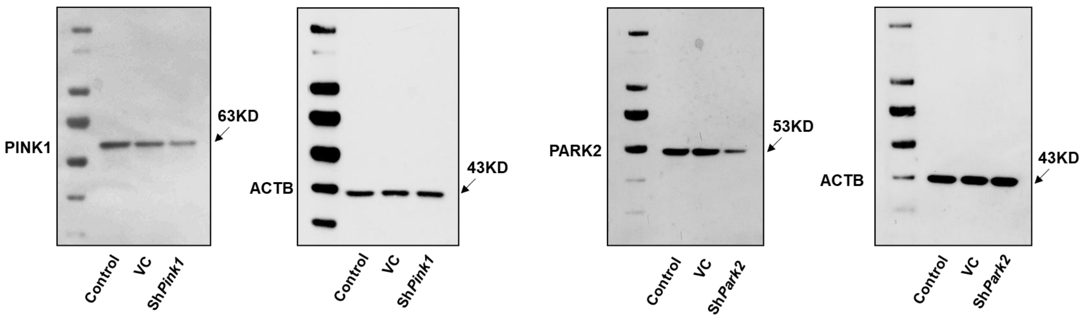

Figure. 5b

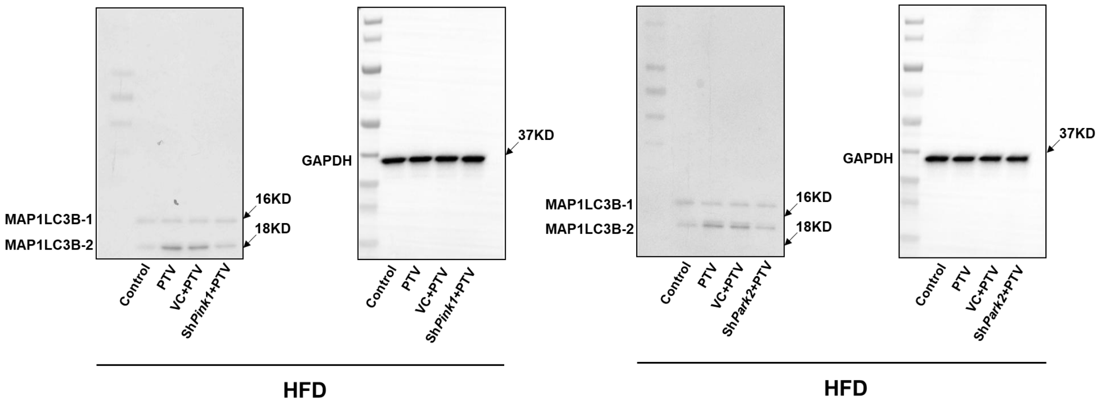

Figure. 6c

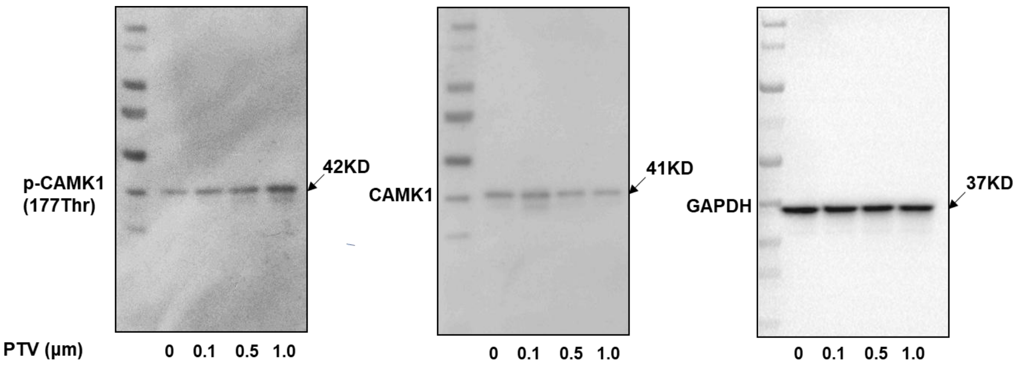

Figure. 7a

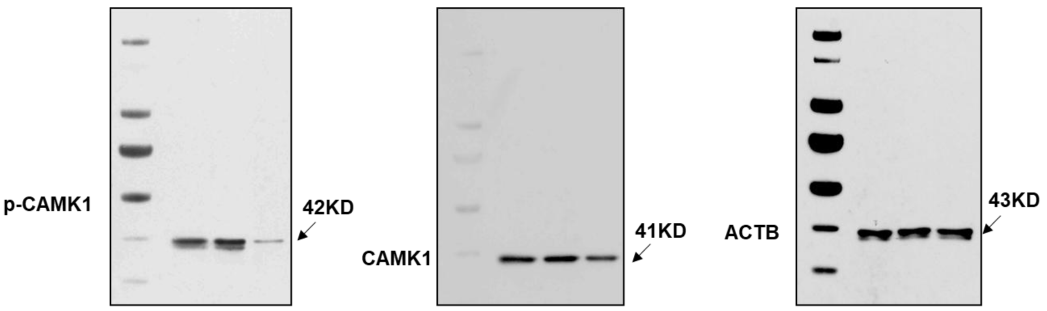

Figure. 7b

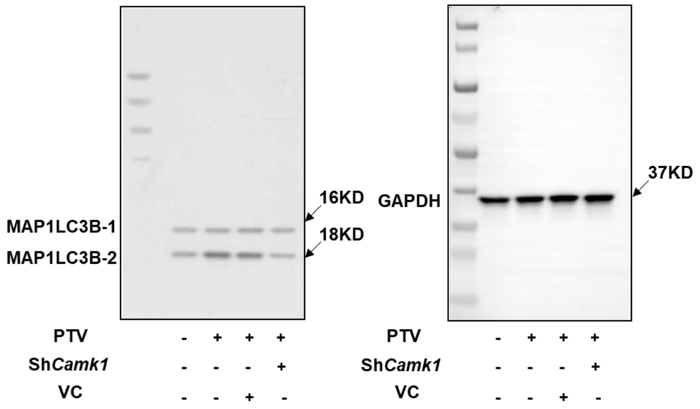

Figure. 7d

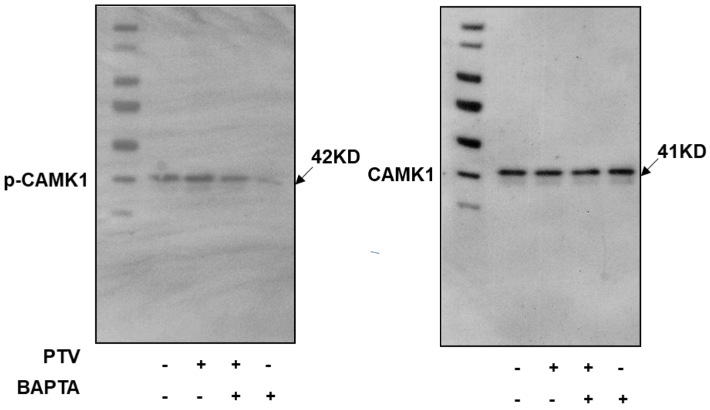

Figure. 7f

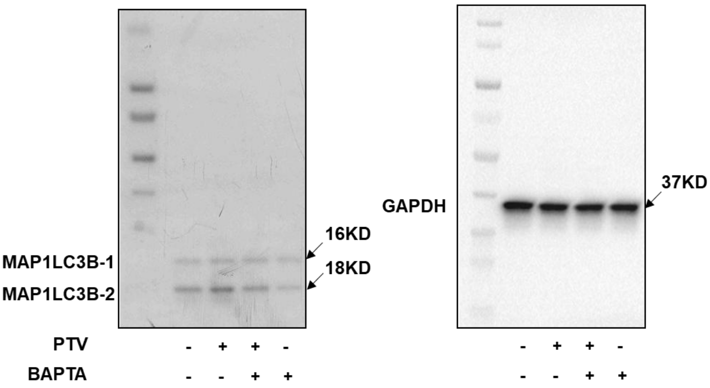

Figure. 8a

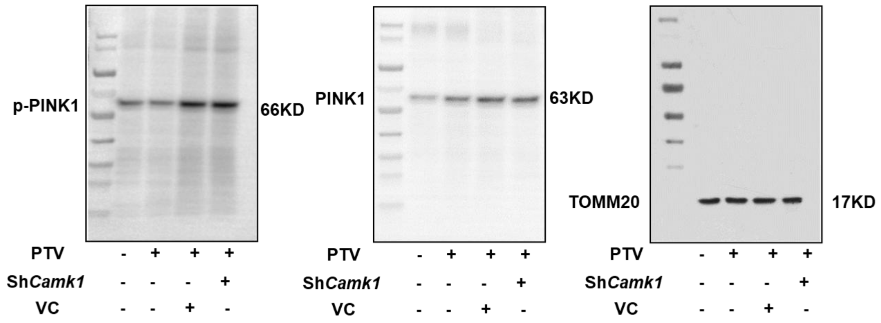

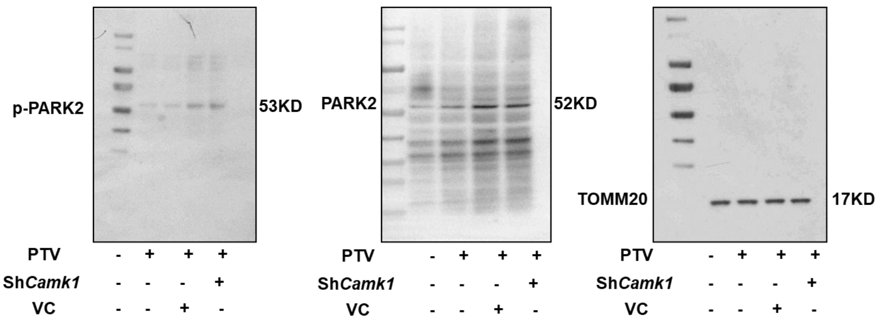

Original Western-Blot Scans (Supplement)

Supplementary Figure. 6b

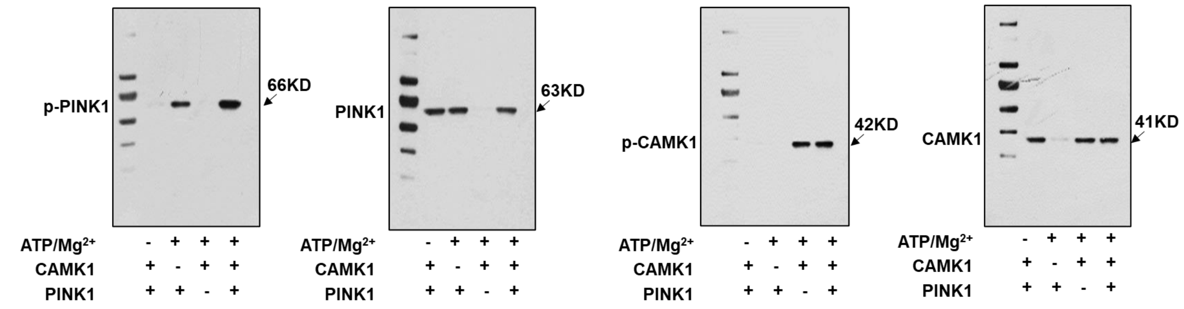

Supplement: Supplementary file 2 — Supplementary Information [file 42003_2022_3081_MOESM2_ESM.pdf]
