# Supplementary material for: Pitavastatin activates mitophagy to protect EPC proliferation through a calcium-dependent CAMK1-PINK1 pathway in atherosclerotic mice
Source: Commun Biol. 2022 Feb 10;5:124. doi: 10.1038/s42003-022-03081-w (PMC8831604; doi:10.1038/s42003-022-03081-w)
Supplement: Supplementary file 3 — Description of Additional Supplementary Files [file 42003_2022_3081_MOESM3_ESM.pdf]

Description of Additional Supplementary Files

File name: Supplementary Data 1

Description: The source data underlying the graphs presented in the main figures.
