# Supplementary material for: Pitavastatin activates mitophagy to protect EPC proliferation through a calcium-dependent CAMK1-PINK1 pathway in atherosclerotic mice
Source: Commun Biol. 2022 Feb 10;5:124. doi: 10.1038/s42003-022-03081-w (PMC8831604; doi:10.1038/s42003-022-03081-w)
Supplement: Supplementary file 5 — Reporting Summary [file 42003_2022_3081_MOESM5_ESM.pdf]

nature portfolio

Corresponding author(s): Huang Lan

Last updated by author(s): Oct 4, 2021

Reporting Summary

Nature Portfolio wishes to improve the reproducibility of the work that we publish. This form provides structure for consistency and transparency in reporting. For further information on Nature Portfolio policies, see our Editorial Policies and the Editorial Policy Checklist.

Statistics

For all statistical analyses, confirm that the following items are present in the figure legend, table legend, main text, or Methods section.

n/a	Confirmed	
☐	☒	The exact sample size (*n*) for each experimental group/condition, given as a discrete number and unit of measurement
☐	☒	A statement on whether measurements were taken from distinct samples or whether the same sample was measured repeatedly
☐	☒	The statistical test(s) used AND whether they are one- or two-sided<br>*Only common tests should be described solely by name; describe more complex techniques in the Methods section.*
☒	☐	A description of all covariates tested
☐	☒	A description of any assumptions or corrections, such as tests of normality and adjustment for multiple comparisons
☐	☒	A full description of the statistical parameters including central tendency (e.g. means) or other basic estimates (e.g. regression coefficient) AND variation (e.g. standard deviation) or associated estimates of uncertainty (e.g. confidence intervals)
☐	☒	For null hypothesis testing, the test statistic (e.g. *F*, *t*, *r*) with confidence intervals, effect sizes, degrees of freedom and *P* value noted<br>*Give P values as exact values whenever suitable.*
☒	☐	For Bayesian analysis, information on the choice of priors and Markov chain Monte Carlo settings
☒	☐	For hierarchical and complex designs, identification of the appropriate level for tests and full reporting of outcomes
☒	☐	Estimates of effect sizes (e.g. Cohen's *d*, Pearson's *r*), indicating how they were calculated

Our web collection on statistics for biologists contains articles on many of the points above.

Software and code

Policy information about availability of computer code

Data collection	Software used include: LAS X software (Leica), Image-Pro Plus 5.0 (Media Cybernetics) and FlowJoTM v10.6.1 software..
Data analysis	Mitophagy analysis was performed using ImageJ. All statistics analyses were performed using SPSS 19.0 software.

For manuscripts utilizing custom algorithms or software that are central to the research but not yet described in published literature, software must be made available to editors and reviewers. We strongly encourage code deposition in a community repository (e.g. GitHub). See the Nature Portfolio guidelines for submitting code & software for further information.

Data

Policy information about availability of data

All manuscripts must include a data availability statement. This statement should provide the following information, where applicable:

- Accession codes, unique identifiers, or web links for publicly available datasets
- A description of any restrictions on data availability
- For clinical datasets or third party data, please ensure that the statement adheres to our policy

The datasets generated during and/or analysed during the current study are available from the corresponding author on reasonable request.

Field-specific reporting

Life sciences study design

All studies must disclose on these points even when the disclosure is negative.

Sample size	Sample sizes were similar to generally employed in the field.
Data exclusions	No data were excluded from the analyzes.
Replication	We confirmed that all attempts at replication were successful.
Randomization	All cell samples were randomly allocated into experimental groups.
Blinding	The binding was not relevant to our basic research.

Reporting for specific materials, systems and methods

We require information from authors about some types of materials, experimental systems and methods used in many studies. Here, indicate whether each material, system or method listed is relevant to your study. If you are not sure if a list item applies to your research, read the appropriate section before selecting a response.

Materials & experimental systems

n/a	Involved in the study	
☐	☒	Antibodies
☐	☒	Eukaryotic cell lines
☒	☐	Palaeontology and archaeology
☐	☒	Animals and other organisms
☒	☐	Human research participants
☒	☐	Clinical data
☒	☐	Dual use research of concern

Methods

n/a	Involved in the study	
☒	☐	ChIP-seq
☐	☒	Flow cytometry
☒	☐	MRI-based neuroimaging

Antibodies

Antibodies used	Antibodies against mouse MAP1LC3B (2775S), SQSTM1 (5114S), PINK1 (6946S), MFN2 (9482S), ATG7 (8558S), BNIP3L/NIX (12396) were purchased from Cell Signal Technology. Antibody for PINK1 (ab23707), PARK2 (ab77924), TOMM20 (ab186734), phospho-Thr177-CAMK1 (ab62215), CAMK1 (ab68234), AMPK (ab32047) and phospho-Thr172-AMPK (ab133448) were obtained from Abcam. Antibody for phospho-Ser65-PARK2 (bs-19882R) was purchased from Bioss. Antibody for phosphor-Ser228-PINK1 (AF7081) was obtained from Affinity Biosciences. Antibodies for flow cytometry test were as follows: APC anti-mouse CD64 (139305, Biolegend), BV421 anti-mouse CD16/32 (101332, Biolegend), PE anti-mouse c-Kit (12-1171-82, ebioscience), PE-Cy7 anti-mouse CD41 (25-0411-80, ebioscience), FITC anti-mouse CD127 (11-1271-81, ebioscience), FITC anti-mouse CD93 (11-5892-81, ebioscience).
Validation	All antibodies used in this study have been extensively validated in previous studies. The specificity, sensitivity, and reproducibility of main primary antibodies (PINK1 and PARK2) were validated in related applications and sample type. We retested the sensitivity with known amount of purified protein as positive controls by western blot analysis. The specificity of PINK1 antibody was assessed in Pink1 knock out cell lines, which is acknowledged as unspecific binding.

Eukaryotic cell lines

Policy information about cell lines

Cell line source(s)	Bone marrow was harvested from the tibias as well as femurs of mice. Bone marrow-derived mononuclear cells (BMNCs) were isolated by density-gradient centrifugation.
Authentication	Ioslated BMNCs were immunostained with DiI-acLDL and FITC-labeled UEA-I lectin. The treble-positive cells were considered as EPCs.
Mycoplasma contamination	The cell lines we isolated were negative for mycoplasma contamination.
Commonly misidentified lines (See ICLAC register)	No commonly misidentified cell lines were used in the study.

Animals and other organisms

Policy information about studies involving animals; ARRIVE guidelines recommended for reporting animal research

Laboratory animals	Male ApoE-/- mice and male C57BL/6J (8 or 16 weeks age, Beijing, China) were used in our study.

Wild animals | The study did not involve wild animals.

Field-collected samples | The study did not involve samples collected from the field.

Ethics oversight | All animal procedures were approved by the Experimental Animal Ethics Committee of the Third Military Medical University before performing the study and conformed to the regulations of Guide for the Care and Use of Laboratory Animals (8th edition, National Research Council, USA, 2011).

Note that full information on the approval of the study protocol must also be provided in the manuscript.

Flow Cytometry

Plots

Confirm that:

- [x] The axis labels state the marker and fluorochrome used (e.g. CD4-FITC).
- [x] The axis scales are clearly visible. Include numbers along axes only for bottom left plot of group (a 'group' is an analysis of identical markers).
- [x] All plots are contour plots with outliers or pseudocolor plots.
- [x] A numerical value for number of cells or percentage (with statistics) is provided.

Methodology

Sample preparation | Bone marrow was harvested by flushing the femurs and filtered through 40 μm filters. BMNCs were isolated using density-gradient centrifugation followed by washing 3 times in PBS. Next, collected cells were stained with the mixed antibodies for flow cytometry. After staining with the listed markers for 45 min in the dark at 4 ℃, samples were washed twice, and transferred to a cell strainer to obtain a single-cell suspension.

Instrument | Beckman Gallios.

Software | FlowJoTM v10.6.1 software.

Cell population abundance | We only analyzed the proportion of bone marrow cells by flow cytometry, but not selected the post-sort fractions to determine their purity.

Gating strategy | Bone marrow cells were firstly gated based on FSC/SSC gate together with the exclusion of c-Kit negative cells. Each gate was further analyzed for the expression of CD41, CD127, CD93 and CD64. Double positive cells (c-Kit+ CD41+) were identified as CMP. c-Kit+ CD64+ cells were identified as GMP. c-Kit+ CD127+ CD93+ cells were identified as CLP.

- [x] Tick this box to confirm that a figure exemplifying the gating strategy is provided in the Supplementary Information.
